# NEI VFQ-25 questionnaire is an excellent option to evaluate the quality of life of Brazilian patients with cataract?

**DOI:** 10.5935/0004-2749.20200103

**Published:** 2024-02-11

**Authors:** Luisa Fioravanti Schaal, Roberta Lilian de Sousa Fernandes Meneghim, Antonio Carlos Lotelli Rodrigues, Carlos Roberto Padovani, Hélio Rubens de Carvalho Nunes, Silvana Artioli Schellini

**Affiliations:** 1 Ophthalmology Department, Faculdade de Medicina de Botucatu, Universidade Estadual Paulista “Júlio de Mesquita Filho”, Botucatu, SP, Brazil; 2 Biostatistic Department, Instituto de Biociência de Botucatu, Universidade Estadual Paulista “Júlio de Mesquita Filho”, Botucatu, SP, Brazil; 3 Research Support Department, Faculdade de Medicina de Botucatu, Universidade Estadual Paulista “Júlio de Mesquita Filho”, Botucatu, SP, Brazil

Dear Editor,

The quality of life of patients with cataract related to vision can be measured using the National Eye Institute Visual Function Questionnaire (NEI VFQ), questionnaire, an instrument developed by the National Eye Institute, USA, and already validated for the Portuguese language^(1)^. The NEI VFQ-25 contains 51 items related to general health, general vision, ocular pain, near and distance activities, social functioning, mental health, role difficulties, dependency, driving, color, and peripheral vision, and it is possible to use the general health information as a predictor of mortality in population studies^(2)^.

We applied the NEI VFQ-25 to evaluate the quality of life of 417 patients with cataract before their surgery, excluding patients with an intellectual level that would preclude comprehension of the questionnaire (dementia or psychiatric disorders), hearing disorders, or refusal to participate in the study. Participants were living in small cities, located in the southwest region of Sao Paulo State, Brazil. The mean age of the participants was 60.1 ± 10.7 years, comprising 243 males (58.3%) and 174 females (41.7%). Most were retirees who were manual laborers for the better part of their work life (74.9%). Most females were housewives without an occupation. Only 6.1% of the study sample worked in intellectual activities. Most patients had poor education and their socioeconomic status was considered medium to low. A small portion of the study population drove, with little or no chance of owning a vehicle. Therefore, only 106 (25.6%) patients could answer the driving component of the questionnaire [20 females (11.5%) and 86 males (35.4%)], which limits the evaluation of the driving component of the survey. Females scored lower in all areas except color vision. In addition, all issues related to social activities were compromised because the cities did not have availability of social venues, such as theaters or cinemas, having little chance of an active social life.

Therefore, the low socioeconomic and cultural levels would likely negatively influence the responses to this validated questionnaire. However, this is the profile of our studied population.

Although our region has an appropriate human development index, poor educational infrastructure and poor personal development levels could negatively influence the outcomes. Because of this, we believe the scores obtained using the NEI VFQ-25 questionnaire might not be representative of the burden of cataract in this population.

In conclusion, the NEI VFQ-25 questionnaire that evaluates the quality of life of patients with cataract is not appropriate for the low-income Brazilian population. Hence, a more adequate questionnaire reflective of the regional conditions might provide an accurate assessment of the burden of cataracts in this region.
